# The state of infectious disease training in Germany before introduction of the new board certification in internal medicine and infectious diseases: past experience and future expectations

**DOI:** 10.1007/s15010-023-02033-8

**Published:** 2023-04-17

**Authors:** Jenny Bischoff, Viktoria Schneitler, Wiebke Duettmann, Andre Fuchs, Sophie Schneitler

**Affiliations:** 1 Department of Internal Medicine I, University Hospital Bonn, Venusberg Campus 1, 53127 Bonn, Germany; 2 Institute of Medical Microbiology and Hygiene, University Hospital Duesseldorf, Moorenstr. 5, 40225 Duesseldorf, Germany; 3 Clinic for Pneumology and Allergology, Center of Sleep Medicine and Respiratory Care, Institute of Pneumology at the University of Cologne, Bethanien Hospital, Aufderhöher Str. 167, 42699 Solingen, Germany; 4 Berlin Institute of Health, Anna-Louisa-Karsch-Straße 2, 10178 Berlin, Germany; 5 Charité - Universitätsmedizin Berlin, Charitéplatz 1, 10117 Berlin, Germany; 6 Internal Medicine III - Gastroenterology and Infectious Diseases, University Hospital of Augsburg, Stenglinstr. 2, 86156 Augsburg, Germany; 7 Institute of Medical Microbiology and Hygiene, Saarland University, Kirrberger Str. 100, 66421 Homburg/Saar, Germany

**Keywords:** Questionnaire, ID, Fellowship, Residency, Training, Survey

## Abstract

**Purpose:**

Recently, the German Medical Association introduced a new board certification in Internal Medicine and Infectious Diseases (ID). Accompanying, current experience with ID training and expectations for the new curriculum were assessed.

**Methods:**

After the development of a digital survey covering four main areas with 59 questions, it was distributed via the German Society for Infectious Diseases (DGI) and other networks following a snowball principle. Participation was carried out digitally in a web-based application.

**Results:**

Between December 2021 and February 2022, 300 datasets were included. 38.9% (114/293) of respondents had completed the additional training in ID. Of those, 54.0% (61/113) were concerned about recognition of previous training certification in the future after the establishment of the new sub-specialization. Overall, 78.5% (135/172) of respondents were satisfied or rather satisfied with the qualification gained through their training, but 8.7% (15/172) felt poorly prepared by their ID training. With regard to the inclusion of microbiology or antimicrobial stewardship (AMS) training into the new ID training curriculum, 84.6% (254/300) and 87.7% (263/300) of participants, respectively, desired an integration. Only 30.8% (53/172) felt sufficiently supported by their employer regarding childcare and 51.7% (89/172) reported missing support for scientific commitment.

**Conclusion:**

Overall, ID training in Germany seems satisfactory so far, but there is uncertainty about future recognition. Participants find that AMS and microbiology training should be integrated into new ID training curricula. New concepts regarding the compatibility of childcare and career as well as the support of scientific commitment seem essential to attract young professionals to the field.

**Supplementary Information:**

The online version contains supplementary material available at 10.1007/s15010-023-02033-8.

## Introduction

To date, in Germany certified infectious diseases (ID) training according to federal standard medical training regulations was available only as an additional training after specialization in, e.g. internal medicine by certification of federally organized regional medical associations. The basic conditions for certified additional ID training are the title as medical specialist of another medical field and at least 12 months of ID training supervised by an appointed instructor. Of those, 6 months must be completed in inpatient or outpatient ID service and an additional 6 months may be served in a related field such as Infection Control and Prevention or microbiology. An independent sub-specialization in ID as a separate internal medicine focus, as is common in Germany for cardiology or gastroenterology, for example, had not been established yet. For this reason, the German Society for Infectious Diseases (*Deutsche Gesellschaft für Infektiologie*, DGI) introduced its own professional designation, the “Infectious Diseases Specialist (DGI)” in 2002, in order to enable further training in ID in Germany that is comparable with international standards. To obtain this certificate, the candidate must be a member of the DGI, have been a specialist in another field (e.g. internal medicine) for at least three years and have worked in an ID or a related field for at least three years. If these three years of employment have not taken place at an ID department certified by the DGI (so-called DGI center), it is necessary to achieve 250 ID-specific continuing medical education points (iCME) of the German ID Academy within 5 years. A detailed description of the further training options can be found in Table S1 in the supplements.

Apart from the additional training in ID certified by the regional medical associations, a further possibility for a nationwide uniform certification of ID training was created in this way. This opportunity for further training was frequently exploited at DGI centers, which are able to cover a certain standard of ID provision and care. Naturally, this has also led to an aggregation of additional training opportunities in ID at DGI centers. Of note, it is also possible to achieve the DGI ID specialist title, even if one is not working at a DGI center by extracurricular activities. The only federal state that already had a board certification in ID for several years is Mecklenburg-Vorpommern. Regarding such differences at federal and state levels, it has to be said that the state level only makes a recommendation, and the practical implementation takes place at the federal level.

At the latest with the outbreak of the coronavirus diseases 2019 (COVID-19) pandemic, it became obvious that ID specialists play an important role in patient care in modern medicine in Germany. In 2015, there were about 500–600 ID specialists in Germany, with about 300 of them providing direct patient care [1]. Approximately, 40 physicians complete ID training in Germany each year [1]. Even before the COVID-19 pandemic, an intensification of ID training has been demanded for years in order to meet the existing need [1, 2]. At least partially, the call for an independent sub-specialization in ID was intended to raise the attractiveness of further training in ID and to attract young medical professionals to the field [3].

Finally, at the 124th German Medical Congress in May 2021 an independent sub-specialization in internal medicine and ID was approved [4]. With the recent revision of the federal standard medical training regulations, the introduction of this new ID sub-specialization training program has been initiated but has not yet been implemented or approved by all federally organized regional medical associations.

In this time of transition, the Young Professionals section (“Young DGI”) of the DGI conducted a survey among its members mostly consisting of ID residents and specialists, and further interested persons. The survey’s goals were to assess past and current experiences, and expectations and desires for the future of training in ID.

## Materials and methods

In peer review discussions, four relevant areas were identified concerning training in infectious medicine: Current training and satisfaction, compatibility of family and career, opportunities for science and research, aspirations and expectations for the new specialization and the future curriculum. These areas were covered with 59 questions (46 multiple-choice questions, six multiple-select questions, five open questions and two grading questions [scale 1–6]). Depending on the training status and response, different questions were presented for answering through logical linkage.

The questionnaire was created as a voluntary anonymous survey using Microsoft Forms (Microsoft Corp., released 2016, Redmond, WA, USA). Consent to data publication had to be confirmed beforehand by the participants.

The questionnaire was evaluated during a pre-test phase. Therefore, the survey was passed on to three colleagues randomly selected from each educational group (student, resident or ID specialist), and asked to complete the survey in advance and to look out for and report any errors or inconsistencies in terms of content and form. The survey was then reviewed and finalized.

The primary call for participation in the survey was distributed among members of the Young DGI via digital networks (email and social media channels) and promoted as a survey of ID training in Germany. In addition, every recipient of the call for participation was requested to forward the survey as often as possible to all potentially interested persons at their own discretion, following the spirit of a snowball principle. No further selection of participants was made, and no one was excluded from completing the survey. No restrictions regarding age, educational status, or other aspects were integrated.

To classify the results, university locations with professorships for infectiology, DGI centers, trainers for infectiology and the current medical statistics of the German Medical Association were determined by means of relevant websites and considered in relation to the study data.

### Statistical analysis

Statistical analysis was performed using IBM SPSS Statistics for Windows (IBM Corp. Released 2020, Version 27.0. Armonk, NY, USA). Continuous variables were summarized as mean or median ± standard deviation, and categorical variables were presented as number and percentage. Comparisons of study cohort characteristics were performed via 2-sided *t*-tests and nonparametric Mann–Whitney-*U* test for values not normally distributed for continuous variables and *χ*^2^ tests (Pearson and Fisher’s exact test) for categorical data. A one-way analysis of variance (ANOVA) was performed for comparisons of continuous variables of at least two independent samples for parameters normally distributed and for parameters not normally distributed a non-parametric Kruskal–Wallis Test was performed. Differences were considered significant at *p* < 0.05 with a confidence interval (CI) of 95%.

## Results

**Fig. 1:**
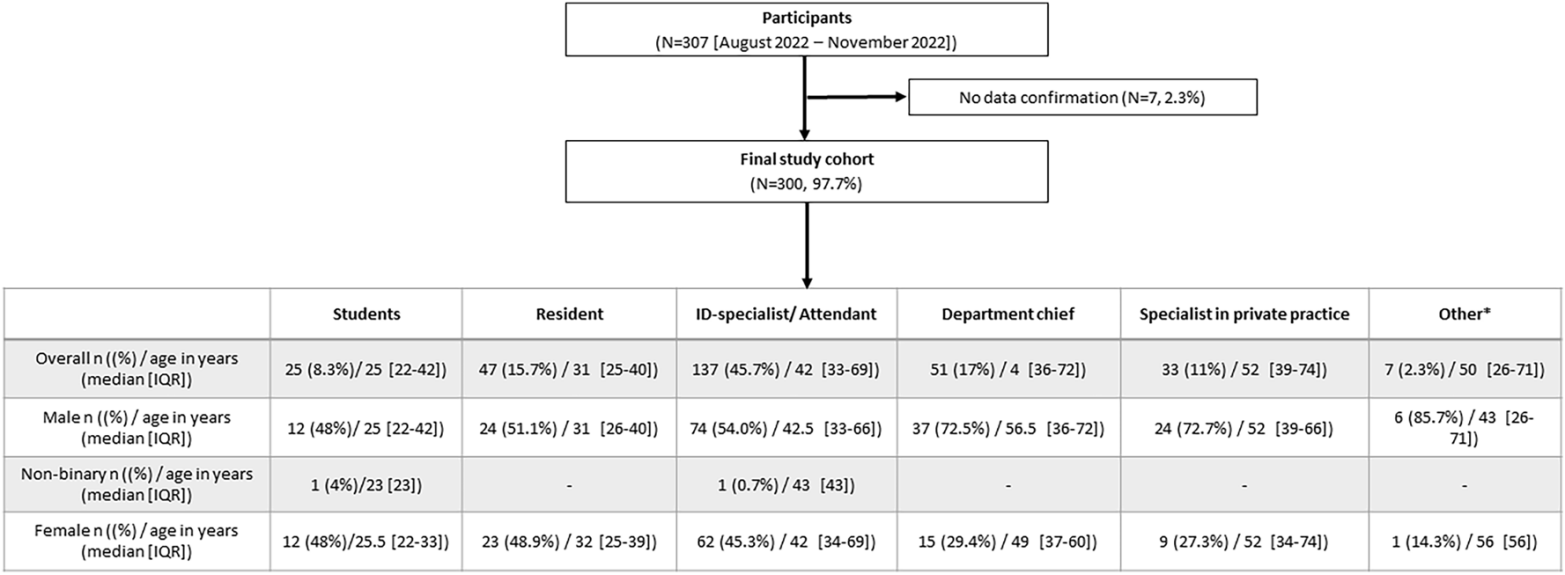
Study flow chart (* other = newly graduated, retired, in private employment, science)

Between December 2021 and February 2022, 307 participants voluntarily completed the survey. Seven participants did not confirm their consent to publish the data and were therefore excluded from the analysis (Fig. 1).

### Study population

In the total study cohort, the median age was 42 years (IQR 22–72) and more males participated (59.0%, Fig. 1). Approximately, one-fourth (24.0%, *n* = 72) of the participants were students or residents. Regarding carrier level, men (71.2%, 37/52) were more albeit not significantly likely to be department chief (*p* = 0.149). The women at this level were approximately younger than the male participants *p* = 0.032, Fig. 1). More men than women worked in private practice (24 vs. 9, *p* = 0.009).

**Fig. 2:**
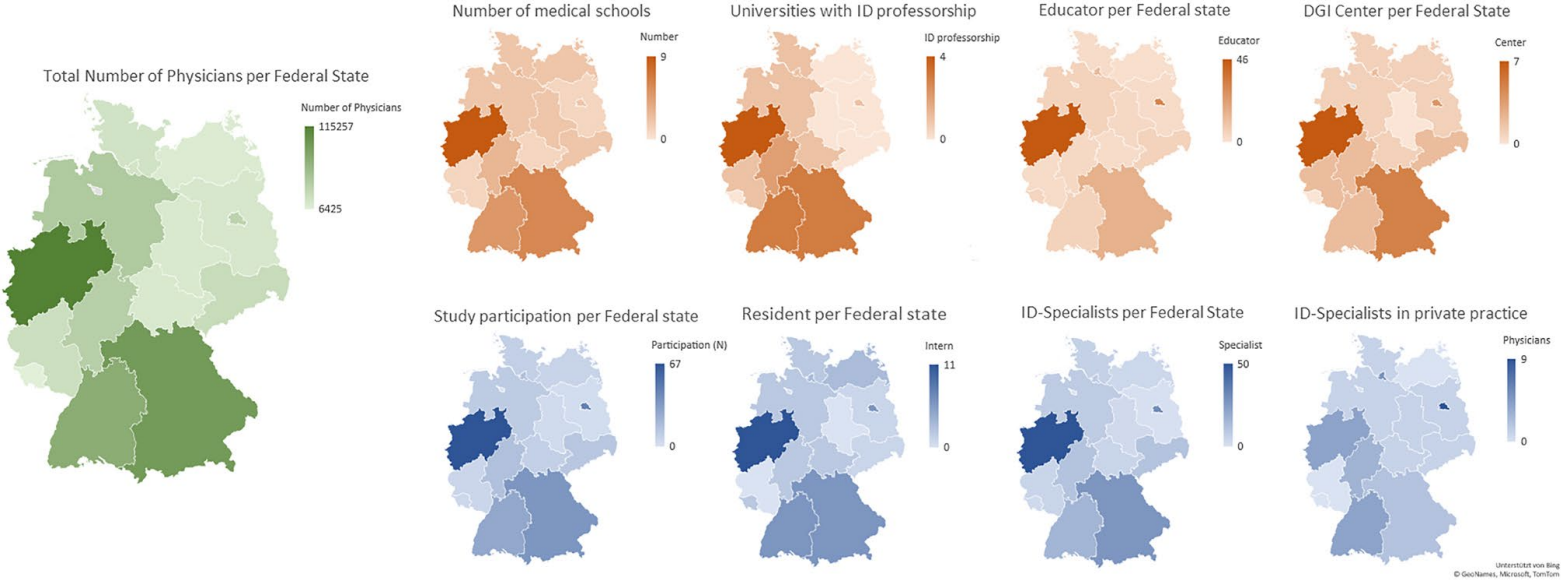
Distribution of physicians in Germany: green depicts the total number of physicians (according to the German Medical Association), brown from left to right: public medical schools, universities with a clinical and occupied professorship in ID, ID educators [additional training and/or sub-specialization] and DGI centers (red, according to the German Medical Association, federal medical associations, DGI information and university website information), and in blue study participants (total number and differentiated according to the level of education and work area [private practice])

The geographic distribution of participants revealed concentrations in some regions such as North Rhine-Westphalia and Berlin (Fig. 2), which however correlate with population density. The comparison with data derived from German Medical Association demonstrates that a representative sample of participants for, e.g. North Rhine and Westphalia-Lippe is depicted. As indicated in the graphical presentation (Fig. 2), the spatial distribution of study participants also follows the uneven distribution of ID specialists and ID training centers across Germany.

### Education and working areas of participants

The results showed that 15.6% (47/300) of the participants were in their residency training and 4.3% (13/300) in the first two years of their training.

**Fig. 3:**
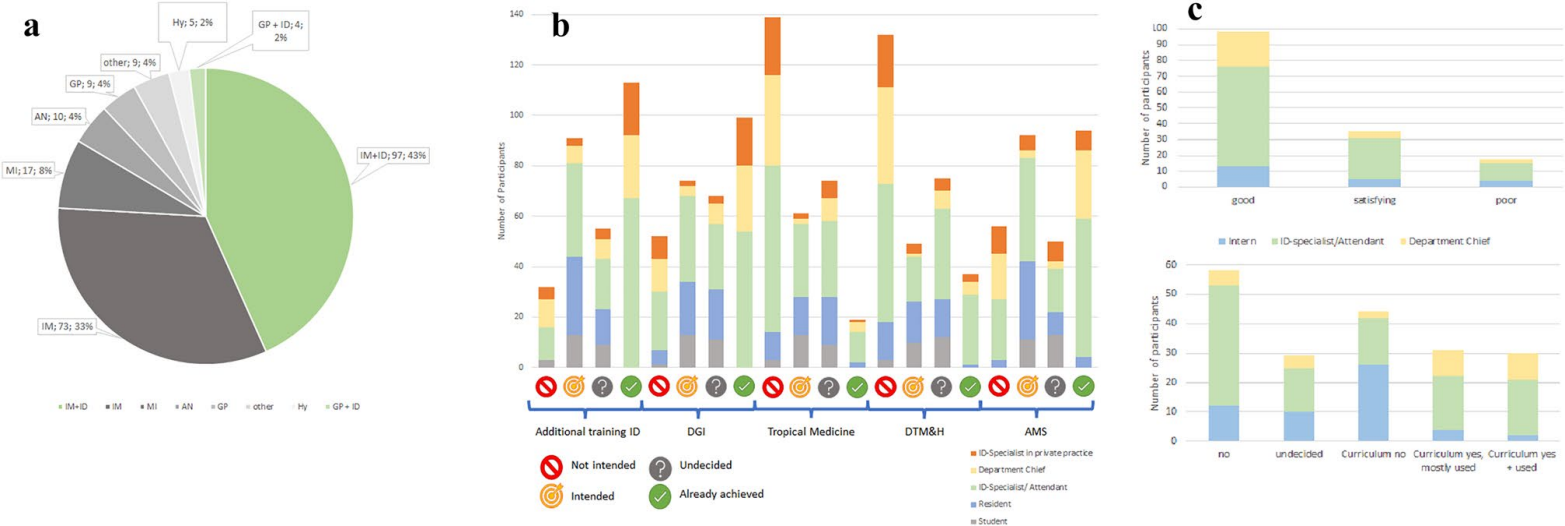
Education and training status, advanced training goals and satisfaction with further training in ID; **a** distribution of specializations among participants, **b** evaluation of the intended and available training paths in infectious medicine and related topics based on the training status (exclusion of others, retired, and research only physicians), **c** satisfaction with additional training in ID training (top), and availability and utilization of curricula in additional training in ID (bottom); *GP* general practitioner, *AN* anaesthesiology, *ID* additional training in infectious diseases, *Hy* infection control and prevention (hygiene), *MI* microbiology/virology, *IM* internal medicine (in the case of double specializations, the first-named specialization was counted); *other: 5 × pediatrics, 2 × laboratory medicine,1 × pharmacology, 1 × gynecology

Out of the 300 participants, 51.3% (*n* = 119) worked at a university hospital with the main focus on patient care (33.7%, *n* = 101) or on science (6.0%, *n* = 18). Furthermore, 18.3% (*n* = 55) of the participants worked at tertiary maximum care hospitals, 13.7% (*n* = 41) at district or municipal hospitals, and 10.7% (*n* = 32) in private practice or in an ambulatory medical care center (3.3%, *n* = 10). The majority of the participating specialists were specialized in internal medicine (76.0%, *n* = 228), many of whom had acquired additional training in ID (Fig. 3).

Roughly one-third of the participants (37.3%, 112/300) aspired to become an ID specialist via the newly developed residency, and 10.7% (32/300) planned to complete a residency in a different specialization, mostly in ID-related fields like microbiology, virology, or Infection Control and Prevention (7.0%, *n* = 21).

Regarding the additional training in ID, 38.9% of the participants (114/293) already completed it and a further 31.4% (92/293) planned to finish it (Fig. 3b). Every tenth participant (10.0%, 29/293) indicated lack of support for completion of the additional training in ID from their workplace.

Currently, additional training in ID is only accessible to specialists in another medical field (e.g. internal medicine). Of those specialists participating in our survey, 50.9% (113/222) had obtained the degree in the frame of the additional training in ID and 44.6% (99/222) had the DGI certification as ID specialist. Both certificates (ID degree and DGI certification) had been obtained by 33.8% (75/222) of specialists. Only in 11.3% (25/222) cases, the DGI certificate alone has been obtained. Residents in comparison to the group of specialists rarely planned to complete the DGI certificate (*p* = 0.002, Fig. 3). Regarding the planned training goals in ID, there was no relevant difference in the group of students (*p* = 0.115, ID specialist versus DGI certificate).

If additional training in ID has already been completed, 22.1% (25/113) of the ID specialists would not choose to redo this training, 18.6% (21/113) would do it again, and 54.0% (61/113) were concerned about the future recognition of the additional training degree, and therefore additionally planned to complete the sub-specialization in ID.

### Interests in related additional training and specialization

Regarding further education in the related field of tropical medicine, nearly half of the participants were not interested in additional training in tropical medicine (47.4%, 139/293) or in the Diploma in Tropical Medicine and Public Health (45.1%, 132/293). On the other hand, 5.1% (15/293) of the participants already completed the additional training and 12.6% (37/293) already completed the Diploma (Fig. 3).

### Personal educational goals

The question whether participants felt well prepared for their future work-life in ID after their ID training was answered by 57.3% (172/300) of them. Of those, 37.2% (64/172) felt that they were well prepared, 41.3% (71/172) felt rather well prepared, and 8.7% (15/172) felt rather poorly prepared, respectively. 12.8% (22/172) of this subgroup could not assess the situation.

### Antimicrobial stewardship and microbiology

With regard to antimicrobial stewardship (AMS), 19.1% of the participants did not plan to attain the certification as “AMS Expert”, 17.1% were indecisive, 31.4% planned to achieve it, and 32.1% already completed certified further training in AMS, respectively (Fig. 3). It became evident that the AMS certificate was less frequently intended in the group of ID specialists in private practice (*p* = 0.019) and among the group of department chiefs (*p* < 0.001) compared to students, residents and ID specialists in general (Fig. 3).

**Fig. 4:**
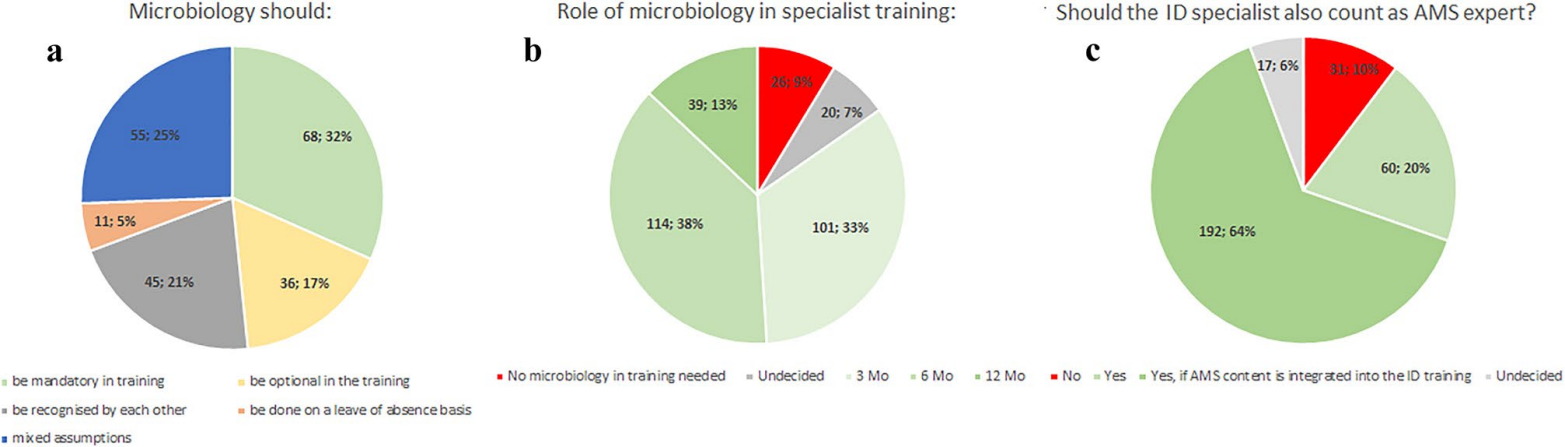
Desired inclusion of microbiology or antimicrobial stewardship in the new ID specialist curriculum; **a** desired integration of microbiology in ID training, **b** duration of desired microbiology training, **c** desired recognition of the ID specialist as an AMS expert; *ID* infectious diseases, *AMS* antimicrobial stewardship

The question whether an ID specialist should in general be recognized as an AMS expert was answered with yes in 20.0% of cases, with no in 10.3% and 64.0% of them answered with yes in case the content of AMS courses would be integrated into the specialist training in ID, respectively (Fig. 4).

With regard to whether training in microbiology and virology (from here on summarized to microbiology) should be included in the new ID specialist curriculum, a total of 84.6% (254/300) would prefer an inclusion of at least 3 to a maximum of 12 months (Fig. 4).

### Compatibility of family and career

**Fig. 5:**
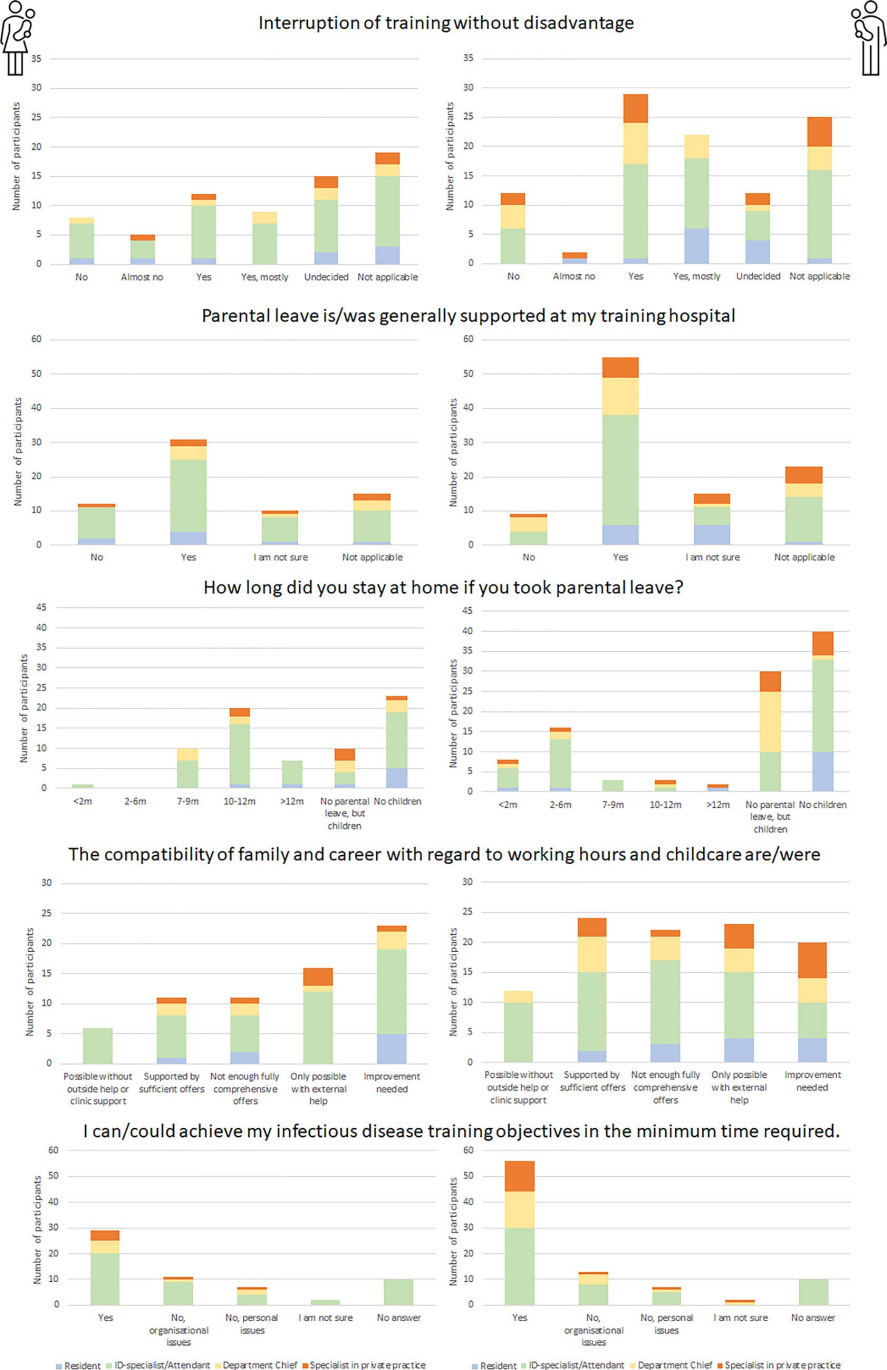
Information on issues of reconciling work, family and support according to gender and training levels

About compatibility of family and career: only 30.8% (53/172) of participants reported that they were sufficiently supported by their employer or that it was possible to take care of their child/children without outside help or support from the employer (Fig. 5). On the other hand, 19.8% (34/172) of participants felt that their employer’s support would not reach far enough and was only accessible for a few persons, whereas 23.3% (40/172) and 26.2% (45/172) of the participants needed help from family members, friends or child-care professionals, or think that the compatibility can be improved considerably, respectively.

Approximately one-third (36.3%) of participants stated to be a parent and reported on parental leave. Of those, significantly more women had taken parental leave (*p* = 0.005, Fig. 5). This difference was most evident in the subgroup of department chiefs. Here, only 21.0% of men and 40.0% of women decided to take parental leave (*p* = 0.024, Fig. 5).

### Compatibility of science and clinical work

Almost two-thirds of the participants (62.0%, 186/300) aspired to work in research although in 12.0% (36/300) the employer would not expect a scientific engagement. 26.0% (78/300) do not want to work in research and 2.7% (8/300) of the participants even though their employer required them to do so. 12.0% (36/300) were indecisive. The scientific commitment was supported for example with granted time off (fully or mostly) in 51.7% (89/172) of cases. On the contrary, their employer mostly not or never supported 26.7% (46/172) of the participants. 20.9% (36/172) by a senior scientist. 62.8% (108/172) of the participants were supervised by a senior scientist most of the time or always, but supervision was lacking in 20.9% (36/172). No significant difference between men and women was found regarding support for research (data not shown). Encouragement of scientific research was equally distributed in DGI centers and non-DGI centers (*p* = 0.677) as well as predominantly perceived as appreciated by residents and specialists/department chiefs (*p* = 0.303). Likewise, sufficient supervision by experienced scientists was reported equally in both subgroups.

## Discussion

This paper reports on a cross-sectional survey that examined how ID training was conducted in Germany before the ongoing transition of the current training standards. The survey captured past experiences with ID training, and expectations and desires for how the training should be shaped in the future.

The survey’s study population mainly comprised clinicians working at university hospitals, tertiary hospitals, and ID centers, which is in line with the organization of ID care and training in Germany. Medical students, residents and specialists in private practice had proportionally low representations in the survey, which might possibly be explained by a lower level of involvement in the Young DGI section and the fact that the survey was distributed within individual networks of participants (Fig. 1). Hence, it seems possible that these groups were less frequently reached by the call for survey participation. Nevertheless, a representative sample was found for some federal states so that a reliable opinion can be assumed overall (Fig. 2).

The majority of the survey participants were internal medicine specialists or internal medicine specialists with additional training in ID, with more than 75% of the participants belonging to this group. Most of the participants had either completed the certified additional training in ID or aimed to complete it, indicating a high acceptance of ID training among participating colleagues. The participation of many colleagues who have already completed ID training or even supervise it themselves is a strength with regard to the evaluation of the current ID training, as they have a very good insight into existing structures. On the other hand, due to the lower participation of younger colleagues, we cannot be certain to have comprehensively assessed their wishes and needs for ID training. However, it could be an indication that networking and interest in contributing to the improvement of the training are not yet very prevalent in this group and should be promoted.

Notably, almost every tenth participant was interested in further qualification in ID, despite their employer not supporting such training. This fact should be seen as an opportunity to attract residents interested in further training to ID centers. Almost one in five would rather opt for a specialist with sub-specialization than for additional training in ID during further training, probably because the new sub-specialization in ID is seen as the more desirable degree for the high proportion of internal medicine specialists among the survey participants. The new specialist training offers the direct and in-depth acquisition of the residency in ID sub-specialization.

The DGI ID specialist was not equally distributed, with residents being less interested in completing the professional designation. Students were equally likely to be interested in completing additional training in ID and the professional designation, suggesting that students might be not fully informed about the different training opportunities, the current status and therefore unable to clearly discriminate between them. Correspondingly, Schneitler et al. showed in a survey of medical students and young doctors that knowledge about possibilities for postgraduate training paths should be generated at the university level to attract for special disciplines [5]. In conjunction with the available data, this shows that interest and possible training paths in the discipline should already be promoted at the university level. Therefore, efforts should be made to improve the visibility and accessibility of relevant training opportunities, especially for students who are interested in pursuing a career in infectious diseases [6]. Residents were probably more likely to be informed about the planned cancellation of the DGI’s ID specialist [7].

The survey found that an overwhelming percentage of participants were committed to research, with many receiving support from senior researchers and off time for science. Moreover, the authors noted that it was no significant difference in the perceived support for scientific involvement between the respondents from recognized ID centers and other hospitals. This observation implies that the quality of support for scientific research and involvement may not be correlated with the institutional status of the hospital or medical center. Instead, the level of support for research and scientific career could be a function of personal factors, such as the mentorship of senior researchers or the availability of research opportunities in the respective hospital or medical center. Further research could shed more light on these factors and help improve the support for scientific career development in the field of infectious diseases. No difference between men and women was found in the research area. It remained unclear why the literature and our data differed here, so this should be addressed in further research [8].

Many participants work at university hospitals and maximum care providers, so the high proportion of people interested in research does not seem unusual. Since research is important for the further development of the discipline, the certified broad support should continue to be granted here and become a fixed component of continuing education.

One in ten ID specialists in the survey reported that he felt he had not received good training to become an infectious disease specialist. Might be, the reason is the missed curriculum at the workplace. In addition to the evaluation of the training institution, this is also to be evaluated with regard to the short training period that was applied in ID training. This fact will certainly be balanced out with the new specialist training so that an increase in training satisfaction can be assumed. Overall, the ID training leaves a good impression with the respondents and different factors not recorded, such as individual support, equipment of the training center, case composition, and support of extracurricular training opportunities could be decisive for this. In any case, this good result should be a reason to undertake efforts to maintain a high level of satisfaction with the quality of ID training in Germany and perhaps to improve it even further.

Even though our survey did not capture the exact family situation of the participants, a large proportion of them are concerned about the compatibility of family, patient care and scientific career. For approximately between 50% (women) and 70% (men) of the respondents, there was either the feeling that their employer did not provide them with sufficient support in balancing work and family or that they had to call on additional private or professional support, e.g. for childcare (Fig. 5). There was no difference between respondents of DGI centers as core training sites and other sites. Not surprisingly, in terms of gender equality this survey also showed that among respondents with children, women had taken parental leave more frequently and for longer time periods than their male colleagues. This difference was particularly striking in the subgroup of department chiefs, who in addition were in any case much more likely to be male. This is in line with the literature already stating that building a family impacts on career opportunities of women [9]. Furthermore, female participants, stated more often that an interruption in further education would have a negative impact on their careers. Regarding the number of female students in medicine these statements show that not only the immediate promotion of young talents is important in order to sustainably invest in the preservation and expansion of a sufficient number of qualified physicians for the field of ID medicine in Germany, but also to promote the compatibility of career and family across gender boundaries [10, 11]. This is even more important because many women, in particular, are lost to the physician's professional life over the course of their careers.

For this purpose, modern working models such as part-time work, parental leave, on-the-job training and similar measures must be introduced into the daily work routine, and disadvantages in further training and career due to parenthood must be reduced. Efforts should be made by educators and employers to increase the compatibility of dedicated clinical and scientific work and family and to prevent an exodus from academic professions to other fields. As in many other occupational groups in Germany men should be encouraged to take parental leave to support the career paths of their partners.

Currently, many regional medical associations are in the process of implementing and introducing a new sub-specialization in internal medicine and ID. Hopefully, this option will soon be available throughout the entire country. A linkage of the new qualification, e.g. with organizational indicators for outpatient or inpatient care is to be expected.

Many of the specialists already decided to train in ID during their studies (31.4%) or during their further training as specialists (34.8%), so that it is clear here that junior staff should be recruited at the early stages of the professional career. Obviously, investment in exciting and dedicated ID education should be seen as an investment into the promotion of new talent in order to attract new physicians to the field of ID [6, 12].

The importance of new ID specialists in Germany should be discussed, as well as the need to integrate training in AMS and microbiology. One argument put forth is that the integration of AMS and microbiology rotations into the training curriculum would be beneficial as both disciplines touch on or include relevant aspects of infectious diseases [13]. Jippes et al. showed that the successful implementation of a new postgraduate training should also take regional factors into account, and here the question of the increasing centralization of infection diagnostics certainly includes that a compulsory rotation can be mapped in a meaningful way [14]. This issue must be addressed critically and constructively, to ensure that the training is not only theoretically valuable but also practical and implementable [13]. Wijk et al. reported that for a successful implementation of a programme it is necessary to form coalitions with others in addition to visions; this is particularly appropriate in the question of microbiology and clinical pharmacy, so that the interdisciplinary exchange is strengthened for all those involved in the project. Further for a successful implementation it is necessary that the educators have enough time and money to create a programme [15, 16].

Overall, the data show that further training in ID was characterized by satisfaction; this is the result of a long-established structure, and the new introduction of the specialist should be as harmonized as possible between the state medical associations. However, attention should also be paid to creating a good interim arrangement between the curricula; the data from Fokkema et al. show that this requires partial support [17].

*This survey has some limitations.* Concerning the representativeness of the data, due to the chosen study method, it cannot be ruled out that mainly thematically interested participants responded, although it could be demonstrated in relation to the Federal Medical Association data that a quantitatively representative sample was certainly achieved in relation to some federal states. The study was carried out on the specialist qualification for internal medicine and ID so this might have influenced the answers with regard to the additive further training.

By distributing the survey via the digital network of the Young DGI section and defining the main thematic focus, a willingly accepted preselection of participants occurred, so that mostly individuals who had at least partially completed their ID training in Germany participated. Also in relation to data published by the German Medical Association in 2021, it is probable that proportionally more senior physicians participated in this survey than are proportionally available in the general medical community, although the specialist specification corresponds to the national level in the ranking [18]. The high participation of already trained ID specialists is both a strength and a weakness of the survey, as they have a good insight into existing structures and curricula. However, the conclusiveness with regard to the wishes and ideas of students and young professionals is limited.

With regard to the field of research education and development, our survey was not addressed to cover fully the compatibility of research and clinical practice. Therefore, it was not possible to sufficiently depict this complex field with a high level of satisfaction.

## Conclusion

The collected data highlight significant uncertainty in the recognition of previous degrees. It is evident that the inclusion of theoretical content, such as AMS, in the curriculum is essential for future specialists. The interdisciplinary nature of infectiology is demonstrated by the desire of many participants to include microbiology in their rotations, despite potential challenges with centralization of infection diagnostic services. It is crucial to discuss interdisciplinary concepts early on to ensure that qualifications are met adequately. Additionally, the study revealed that it is challenging for the younger generation to navigate the various training paths available, emphasizing the need to provide early guidance. Moving forward, it is essential to work towards gender equality in both clinical and private practice settings by improving the balance between family and career. Overall, the survey suggests a generally high level of satisfaction with the quality of ID training in Germany, but efforts should be made to maintain and improve it further.

## Supplementary Information

Below is the link to the electronic supplementary material.

Supplementary file1 (DOCX 19 KB)

## Data Availability

The data that support the findings of this study are not openly available due to reasons of sensitivity and are available from the corresponding author upon reasonable request.
