# Supplementary material for: The state of infectious disease training in Germany before introduction of the new board certification in internal medicine and infectious diseases: past experience and future expectations
Source: Infection. 2023 Apr 17;51(3):589–98. doi: 10.1007/s15010-023-02033-8 (PMC10106872; doi:10.1007/s15010-023-02033-8)
Supplement: Supplementary file 1 — Supplementary file1 (DOCX 19 KB) [file 15010_2023_2033_MOESM1_ESM.docx]

**Supplementary Information**

**Infection**

**The state of infectious disease training in Germany before introduction of the new board certification in internal medicine and infectious diseases - past experience and future expectations**

Jenny Bischoff^*^, Viktoria Schneitler^*^, Wiebke Duettmann, Andre Fuchs^#^, Sophie Schneitler^#^

Corresponding author

Dr. med. Jenny Bischoff

University Hospital Bonn

Department of Internal Medicine I

Venusberg Campus 1, 53127 Bonn, Germany

0228-287-13933

[jenny.bischoff@ukbonn.de](mailto:jenny.bischoff@ukbonn.de)

Table S1 Infectious Disease training in Germany

| **Regulations** | | |
| --- | --- | --- |
| Old | | New |
| DGI | Medical Associations | |
| **Conditions** | | |
| - DGI membership - Specialist certificate for at least 3 years - Three years of work in ID, incl. one to two years in the field of clinical care - 250 ID-specific continuing education points (iCME) of the ID-Academy in a period of 5 years, if the candidate is not working at a DGI center | - medical specialist - 12 months ID training | - 72 months in internal medicine - Of these, 36 months must be completed in internal medicine and ID -24 months in at least 2 other specialist competences - In addition, 6 months each must be completed in the emergency room and intensive care medicine. |

Tab. S1 Infectious Disease training in Germany; Abbreviations: DGI: German Society for Infectious Diseases (Deutsche Gesellschaft für Infektiologie)
